# Enhanced Bioactivity of Cu-Doped Bioactive Glass Coatings on Human Freeze-Dried Cortical Bone: An In Vitro Study

**DOI:** 10.3390/bioengineering12040354

**Published:** 2025-03-29

**Authors:** Silvia Brogini, Matilde Tschon, Leonardo Vivarelli, Alessandro Gambardella, Angela De Bonis, Gianluca Giavaresi, Milena Fini, Dante Dallari, Julietta V. Rau, Marco Govoni

**Affiliations:** 1 Surgical Sciences and Technologies, IRCCS Istituto Ortopedico Rizzoli, 40136 Bologna, Italy; silvia.brogini@ior.it (S.B.); matilde.tschon@ior.it (M.T.); alessandro.gambardella@ior.it (A.G.); gianluca.giavaresi@ior.it (G.G.); 2 Reconstructive Orthopaedic Surgery and Innovative Techniques—Musculoskeletal Tissue Bank, IRCCS Istituto Ortopedico Rizzoli, 40136 Bologna, Italy; dante.dallari@ior.it (D.D.); marco.govoni@ior.it (M.G.); 3 Department of Science, University of Basilicata, 85100 Potenza, Italy; angela.debonis@unibas.it; 4 Scientific Director, IRCCS Istituto Ortopedico Rizzoli, 40136 Bologna, Italy; milena.fini@ior.it; 5 Istituto di Struttura della Materia, Consiglio Nazionale delle Ricerche (ISM-CNR), Via del Fosso del Cavaliere 100, 00133 Rome, Italy; giulietta.rau@ism.cnr.it

**Keywords:** allogeneic freeze-dried cortical bone, bioactive glass, copper, coating, pulsed-laser deposition

## Abstract

Bone grafting is one of the most used surgical techniques to favor bone regeneration and repair in orthopedic procedures. Despite autografting continuing to be considered the gold standard, allogeneic bone tissues remain a viable alternative albeit in the last decades, only a few studies have been carried out to translate enhanced allogeneic bone grafts into clinical solutions. In this in vitro study, cortical allogeneic bone samples were coated with copper-doped bioactive glass 45S5 (Cu-BG) by means of the pulsed-laser deposition technique to combine the mechanical support and osteoconductive properties of human bone with the osteogenic and pro-angiogenic features of the bioactive material. Contact angle (CA), scanning electron microscopy (SEM), and atomic force microscopy (AFM) measurements were carried out to quantitatively compare the impact on the bone surface properties of the morphological changes induced by the presence of the coating. Specifically, the obtained results have shown a total absorption of the drop on the coated samples. The coating on the bone tissue surface consisted of a homogeneous Cu-BG background layer with micrometric grain-like aggregates on it—a morphological feature that can facilitate osteoblast adhesion and proliferation. Cytotoxicity and cell viability were carried out on Saos-2 osteoblast-like cells, demonstrating the biocompatibility of the novel composite bone tissue and the absence of cytotoxic residuals. Moreover, human bone marrow-derived mesenchymal stem cells (hBMSCs) were seeded on Cu-BG and not-coated (NC) samples to evaluate the bioactivity and their differentiation toward the osteogenic phenotype. Our findings showed the pro-osteogenic and pro-angiogenic potential of Cu-BG coatings, although dynamic changes were observed over time. At seven days, the Cu-BG samples exhibited significantly higher expressions of *SP7*, *SPP1*, and *BGLAP* genes, indicating an enhanced early osteogenic commitment. Moreover, *VEGF* expression was significantly increased in Cu-BG compared to the control. These results pave the way for the development of an innovative class of bone-based products distributed by tissue banks.

## 1. Introduction

The global demand for bone grafts and substitutes is significant, with over 2.2 million procedures performed annually [1]. Due to the increasing burden of bone defects in an aging population [2], further research into alternatives to conventional treatments is needed. Allogeneic bone tissues remain a viable alternative to autologous grafts because they avoid pain at the donor site and are available in different shapes and sizes, thus reducing the operating times [3]. Specifically, cortical bone allografts are widely used for orthopedic procedures, including spinal fusion and high osteotomies, owing to the combination of biomechanical competence and biological osteoinductive properties. Moreover, when they are processed by the freeze-drying technique, they maintain the natural structure of the bone and the related mineral and extracellular matrix components (e.g., fibronectin, heparan sulphate, dermatan sulphate, chondroitin sulphate, and hyaluronic acid) [4,5], along with having a longer shelf life compared to fresh or frozen grafts [6]. Nevertheless, there is a notable lack of scientific research aimed at developing innovative bone-based products from cortical bone specimens that ensure high biomechanical competence to be translated into clinical solutions [7,8].

For metallic implants, the deposition of specific bioactive coatings on their surfaces represents a strategy implemented to promote the osseointegration process between bone and implant interfaces, thereby enhancing the life span of the implant and reducing the necessity for revision surgery [9,10,11]. Similarly, tuning specific parameters (e.g., temperature, pressure, deposition rate, and film thickness) to ensure that the biological properties of native hard tissues are not altered, bioactive-coating deposition techniques may be applied to cortical bone tissue surfaces. Based on this rationale, the aim of this study was to apply a nanometric bioactive film to human bone specimens to conjugate the biomechanical performance of cortical bone and boost its angiogenetic properties. In this context, various coatings and deposition techniques can be exploited to identify the most promising candidates for the study protocol. Concerning the variety of available biomaterials (e.g., silver, silicates, calcium phosphates, bisphosphonates, etc.) [11] used for coating of orthopedic implants, bioactive glasses (BGs) are synthetic silicate-based ceramics that have been investigated and used for bone replacement for over 50 years [12]. Among BGs, Bioglass^®^ 45S5 is one of the most widely recognized commercially available bioactive glasses used as bone graft substitutes to treat bone defects in a variety of clinical applications within the field of orthopedics and traumatology, as well as in the fields of cranial and facial surgery [13]. Although biocompatibility and osteoconductivity are well-established properties of Bioglass^®^ 45S5, the modification of the glass composition by adding ions with specific biological functions has prompted researchers to investigate different strategies to improve its in vivo performance [14,15,16].

In this regard, ion-doped bioactive glass 45S5 is employed to create nanoscale thin bioactive films both on resorbable and non-resorbable metallic substrates with the objective of functionalizing medical implants or prostheses [17]. Among the various potential metal ion candidates [18,19], copper (Cu) has emerged as a particularly promising option, garnering increasing interest from the scientific community. This is largely due to its essential role in biological function, including metabolic processes and antimicrobial activities [20]. With regard to the musculoskeletal apparatus, several studies have demonstrated that copper ions (i) encourage osteogenic activity [21], (ii) suppress osteoclast behaviour [22], (iii) play a pivotal role in the synthesis and deposition of a collagen and elastin matrix [23,24], and (iv) promote angiogenesis [25]. For these properties, copper-releasing bioactive glass 45S5 (Cu-BG) was selected as a bioactive biomaterial for the freeze-dried cortical bone coating [26].

Among the various physical vapor deposition techniques involving vaporization (e.g., sputtering [27,28]), atomization (e.g., electron beam evaporation [29] and pulsed-laser deposition—PLD [30,31]), or both (e.g., ion plating [32]), PLD was selected for its capacity to produce thin films with the maintained stoichiometry of multi-element targets during deposition, once the deposition parameters, especially fluence, are optimized [33]. In our previous study, we have shown that, by using the deposition condition reported in this work, it is possible to deposit at room temperature thin films of glass–ceramic materials that retain the target stoichiometry [34]. Although alternative methods, such as chemical vapor deposition (CVD), plasma spraying, or sputtering, may offer advantages in terms of coating uniformity, especially on porous surfaces, they often encounter difficulties in controlling the exact chemical composition during the deposition process. These methods can sometimes lead to deviations from the expected stoichiometry due to problems such as re-evaporation or complex reaction dynamics, or the incorporation of contaminants.

Therefore, the choice of the PLD technique is justified by its ability to preserve chemical integrity, prioritizing the functional properties of the coating over surface uniformity. Furthermore, this technique stands out for its high versatility, with minimal restrictions on the source material [35]. Nevertheless, PLD is typically used to create coatings on synthetic materials, as demonstrated in our previous study, where we investigated the deposition process for coatings using the same target material but with different substrates (Ti foil and Si (100) wafer) [26]. To the best of our knowledge, however, this technique has never been utilized for the fabrication of coatings on biological tissue.

Therefore, in this study, for the first time, PLD was used to produce a nanometric film of Cu-BG on freeze-dried cortical bone tissues. The aim was to develop a multifunctional composite biomaterial that combines the osteoconductive and biomechanical properties of cortical bone with Cu-doped BG. The hypothesis is that the Cu-BG coating would enhance the osteogenic and angiogenetic properties of the allograft. Surface wettability, scanning electron microscopy (SEM), and atomic force microscopy (AFM) measurements were conducted to examine the surface properties of the bone following the deposition and sterilization of Cu-BG. In addition, biological tests were performed on (i) Saos-2 osteoblast-like cells to ascertain the absence of potential cytotoxicity and (ii) human bone marrow-derived mesenchymal stem cells (hBMSCs) to evaluate the composite’s bioactivity in terms of enhancing osteogenic differentiation and angiogenic properties.

## 2. Materials and Methods

### 2.1. Preparation of Cortical Bone Specimens

**Figure 1:**
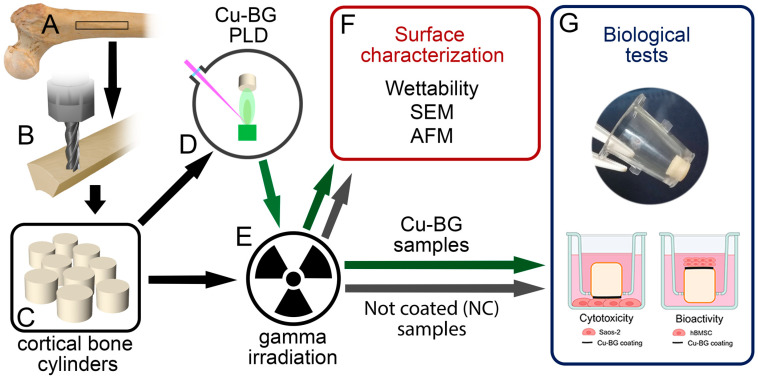
Schematic diagram of sample preparation, treatments, and analyses. Processing of the allogeneic femoral diaphysis starting from a femoral shaft ((**A**): rectangular black box). CNC milling (**B**) was performed on each femoral shaft to obtain 26 cortical bone cylinder samples (**C**) in total. PLD deposition on a part of the samples (**D**). Sterilization by gamma irradiation of all samples (**E**). Surface characterization analyses (**F**). Biological tests (**G**). Schematic representation of the in vitro experimental setup for cytotoxicity and bioactivity assessments and representative image of the Cu-BG-coated material placed inside the transwell insert before exposure to the cell cultures.

A right femur was harvested from a 57-year-old male cadaver donor by the Musculoskeletal Tissue Bank of IRCCS Istituto Ortopedico Rizzoli (Bologna, Italy; EU TE Code: IT000096) during the routine activities of tissue procurement. However, in this study, only specimens not suitable for transplantation and processed for validation procedure purposes were used, according to Italian Legislation and National Transplant Centre’s guidelines. The femur was stored at −80 °C until its processing inside a GMP-Class A environment. After thawing, the femoral diaphysis was sectioned using a band saw (C/E 165, La Minerva, Bologna, Italy) to obtain six cortical bone shafts of 10 cm in length, 9 mm in width, and approximately 6 mm in height (Figure 1A).

Then, the six shafts were clamped within a computerized numerical control (CNC) milling machine (model Bright, Delta Macchine, Rieti, Italy). A milling process was applied (Figure 1B) according to the procedure for custom allograft processing previously reported [36,37]. CNC machining allows for the systematic reproduction of sample parameters set by pre-programmed computer software, thereby avoiding biases related to significant differences in the samples’ shape, volume, and number of seeded cells. Moreover, CNC machining guarantees the worker’s safety, minimizing the chance of hand injuries, especially during the preparation of small samples.

The tool trajectory was pre-programmed with Rhinoceros CAD software (version 4.0, Robert McNeel & Associates, Seattle, WA, USA) with the RhinoCAM plugin (version 2.0, MecSoft Corporation, Irvine, CA, USA). This milling process yielded 26 samples, namely cortical bone cylinders with a diameter of 5–5.5 mm and a height of 4 mm (Figure 1C).

To remove any residual splinters and small defects, and to minimize potential bias, all samples were manually scraped with a fine-grain rasp on both circular surfaces. Then, the samples were cleaned and defatted with organic solvents, washed with sterile water, and freeze-dried (VirTis Genesis 25, SP Scientific, Warminster, PA, USA) by the same protocol applied to allogeneic tissues distributed for transplant purposes.

Of the 26 samples, half underwent PLD for Cu-BG deposition (Cu-BG samples) and half did not (non-coated samples, NC). Finally, the cortical bone samples were treated as shown in Figure 1D,E.

The absence of microbial contamination was confirmed by culture methods performed after the bone tissue procurement and during each tissue processing step within the clean room.

### 2.2. Cu-BG Deposition

Thirteen samples were coated with Cu-BG thin films (~500 nm) using the PLD technique. Cu-BG was produced by melt processing, as described previously [38]. The obtained powder was pressed to get pellets (9 mm diameter, 5 mm height), which were sintered in air (peak temperature 1100 °C, heating rate 5 °C/min, and cooling rate 20 °C/min). Cu-BG has the following composition expressed in wt%: 45% SiO_2_, 24.5% Na_2_O, 6% P_2_O_5_, 19.5% CaO, and 5% CuO. For the PLD experiments, each sample was placed in a stainless-steel vacuum chamber equipped with a scroll-turbomolecular pumping system for a final pressure of 10^−4^ Pa. The Nd:YAG laser source (λ = 532 nm, τ = 7 ns, 10 Hz) was directed into the vacuum chamber and focused on the Cu-BG target by means of a quartz lens with a 350 mm focal plane. The previously optimized experimental conditions for the deposition were the target–substrate distance = 2 cm, deposition time = 4 h (144,000 pulses), and laser fluence = 12 J/cm^2^ [39]. This fluence was achieved using a laser energy of 15 mJ and a spot diameter of 0.1 cm^2^ on the target surface. In our experimental setup, a target-to-substrate distance of 2 cm was selected because it allows for efficient material deposition while preventing the impingement of highly energetic plasma species on the substrate. Specifically, under the experimental conditions employed, namely the chosen laser fluence and wavelength, the plasma plume does not extend sufficiently to “wash” or adversely affect the substrate surface [40]. Physical–chemical analyses of the target material were previously reported [26]. The coated samples were fixed with an inert tape on the surface of a polystyrene 24-well plate.

### 2.3. Sample Sterilization

Since the PLD apparatus is placed in a clean, non-classified area, after deposition, the Cu-BG samples were sterilized by gamma irradiation (Gammatom S.r.l, Como, Italy). However, to avoid biases on the biological and mechanical properties of bone tissues related to gamma ray sterilization [41], both the NC and Cu-BG samples were treated with a nominal dose of 25 kGy, in accordance with ISO 11137-2:2013 [42] (Figure 1E).

### 2.4. Surface Characterization

#### 2.4.1. Wettability

Surface wettability was characterized by water contact angle (CA) measurement (Digidrop CA meter, GBX Instrumentation Scientifique, Romans sur Isère, France). The volume of the drop was ~0.50 μL, corresponding to a contact radius with the surface, uncoated or coated, of ~0.5 mm. CA values were acquired on at least seven different positions over the sample surface, and the mean value was provided.

#### 2.4.2. Scanning Electron Microscopy

Sample morphology was analyzed by SEM (EVO/MA10, ZEISS, Oberkochen, Germany) working at 20 kV and operating in a 10^−6^ mbar vacuum chamber at room temperature.

#### 2.4.3. Atomic Force Microscopy

AFM measurements were performed by an NT-MDT (Moscow, Russia) system equipped with an upright optical microscope. NSG30 tips (NT-MDT, Moscow, Russia) with a resonant frequency around 250 kHz and operating in tapping mode were used. All topographic images were acquired at a 256 × 256-pixel resolution. Root-mean-square (RMS) roughness was extracted from each topography by the open source Gwyddion Software (Brno, Czech Republic) and averaged over 10 non-overlapped images per lateral scale (L) within the 2 µm to 10 µm range.

### 2.5. Biological Tests

The in vitro experimental setup was adopted to assess both cytotoxicity and bioactivity (Figure 1G). Cytotoxicity by direct contact was evaluated using Saos-2 osteoblast-like cells seeded at the bottom of the well, with the Cu-BG-coated material placed in direct contact via a transwell insert. Bioactivity was assessed by seeding hBMSC directly onto the Cu-BG-coated surface. The setup was specifically designed to ensure that the Cu-BG-coated side of the material was in direct contact with the cells, optimizing the evaluation of its biological effects.

#### 2.5.1. Cytotoxicity Evaluation

The tests were performed in accordance with international standard UNI EN ISO 10993-5:2009—“Biological evaluation of medical devices—Part 5: Tests for in vitro cytotoxicity” [43]. A test with direct contact between the experimental materials and the cells was selected at the experimental time of 48 h, as suggested by the ISO guideline. In addition to positive and negative controls, a reference material was used.

Saos-2 osteoblast-like cells (ATCC, HTB-85) preserved in −180 °C liquid nitrogen were thawed and cultured in Dulbecco’s modified eagle’s medium (DMEM, Sigma-Aldrich, Darmstadt, Germany) with 10% fetal bovine serum (Euroclone, Milan, Italy) and 100 IU/mL penicillin–100 μg/mL streptomycin (Sigma-Aldrich, Darmstadt, Germany) at 37 °C in 5% CO_2_ humidified atmosphere. At 80% confluence, cells were detached with trypsin 0.05% (*w*/*v*)/EDTA 0.02% (*w*/*v*) (Sigma-Aldrich, Darmstadt, Germany) and counted in a hemocytometer chamber. A Saos-2 cell suspension at a concentration of 1 × 10^4^ cell/cm^2^ was seeded in 24-well plates for cytotoxicity in vitro tests, respecting the ratio of 1/10 between the area of the materials and the area of cell seeding, as indicated by the standard. The Saos-2 osteoblast-like cell line was selected, taking into account that the materials are finalized to be used in contact with bone tissue. The next day, after checking the correct cell adhesion, three Cu-BG samples and three NC samples were sterilely positioned in contact with the Saos-2 cells. Moreover, three wells for negative control (CTR−) with DMEM only and three wells for positive control (CTR+) with a 1% phenol solution in DMEM were prepared. The plates were incubated for 48 h at 37 °C in a 5% CO_2_ atmosphere. At the end of the experimental time, the supernatant was collected from all wells.

##### Cell Proliferation and LDH Release

To evaluate cell proliferation, a WST1 test (Roche Diagnostics, Manheim, Germany) was performed; 100 μL of WST1 (tetrazolium salt) and 900 μL of fresh culture medium were added to every well and cultures were incubated at 37 °C for a further 4 h. Tetrazolium salt is transformed to formazan by reductase of the mitochondrial respiratory chain, active in viable cells only. Supernatants were measured by a spectrophotometer at 450/625 nm. Cell viability is a fundamental parameter of evaluation, as a reduction in viability by more than 30% is considered a cytotoxic effect. The obtained values were then expressed as a percentage of proliferation over CTR−. To detect LDH release in the supernatant, an enzyme kinetic test (BioChain, Newark, CA, USA) was used; 45 μL of reagent were added to 100 μL of cell supernatant in a 96-well plate. After 30 min of incubation at room temperature (RT) protected from light, the samples were evaluated with a spectrophotometer at 490/655 nm. The LDH dosage is an indirect parameter of cytotoxicity because its release is due to damage to cell membranes. The obtained values were then expressed as a percentage of cytotoxicity according to the following formula:

$$\%LDH\;release=\frac{{[LDH}_{material}]-[{LDH}_{CTR-}]}{{[LDH}_{CTR+}]-[{LDH}_{CTR-}]}$$

##### Neutral Red Staining

The Neutral Red dye is used for a qualitative and quantitative evaluation of cell morphology. Changes in morphology, vacuolization, lysis, and detachment are useful observations for grading cytotoxicity. It is actively absorbed by living cells, so it allows for clearly distinguishing them from dead cells. A 0.033% solution of Neutral Red staining (Sigma-Aldrich, Darmstadt, Germany) in a culture medium, warmed to 37 °C, was added to all wells at the end of the experimental time for a further 120 min. The cultures were examined by light microscopy for the evaluation of cell morphology, and explanatory images were selected. Then, an equal volume of Neutral Red Solubilization Solution was added to each well, and the OD values were measured with a spectrophotometer at 540/655 nm. The results were reported as percentages over CTR−.

#### 2.5.2. Bioactivity Evaluation

##### hBMSC Viability

Human bone marrow-derived mesenchymal Stem Cells (hBMSCs, Cod. PT-2501, Lonza Bioscience, Basel, Switzerland) were expanded in a mesenchymal basal medium (MSCBM Basal Medium, Lonza Bioscience, Basel, Switzerland) complete with the appropriate supplements (MSCGM™ Mesenchymal Stem Cell Growth Medium BulletKit™, Lonza Bioscience, Basel, Switzerland), 100 U/mL penicillin, and 100 μg/mL streptomycin (Sigma-Aldrich, Darmstadt, Germany), for about 14 days in T75 flasks (Corning Cell Culture Flask, Sigma-Aldric, Darmstadt, Germany) in standard conditions. The medium was replaced every two/three days. After reaching confluence, the cells were detached, and 5 × 10^4^ hBMSCs were seeded on each sterile Cu-BG and NC specimen and on polystyrene wells used as control (CTR). The test samples were placed in 24-well plates and incubated for 1 h in standard conditions to enhance cell adhesion. Then, 1 mL of fresh basal medium was added. After 24 h of culture, the growth medium was completely replaced with an osteogenic differentiation medium composed of complete basal medium supplemented with β-glycerolphosphate (10^−4^ M), ascorbic acid (50 µg/mL), and dexamethasone 10^−7^ M. The medium was replaced twice a week. After 7 and 14 days, the specimens of each type were analyzed for hBMSC viability.

The viability of hBMSCs was quantified by an AlamarBlue™ assay (Invitrogen, Waltham, MA, USA) at each experimental time. AlamarBlue™ dye, mixed with culture medium (1:10 *v*/*v*), was added to hBMSCs, seeded onto the samples, and incubated for 4 h in standard conditions. The amount of fluorescence is proportional to the number of living cells and corresponds to the cell’s metabolic activity. The fluorescent product was quantified at 530ex–590em nm wavelengths using a microplate reader (VICTOR X2030, Perkin Elmer, Milan, Italy) and expressed as relative fluorescence units (RFU).

##### hBMSC Osteogenic Differentiation

The differentiation of hBMSCs through an osteoblast phenotype was analyzed by the expression of specific genes (Table 1). Furthermore, *VEGF* expression was analyzed to assess the vasculogenetic capacity.

After 7 days and 14 days, total RNA was extracted from the cells seeded on the samples using the commercial RNeasy Mini Kit (Purelink™ RNA miniKit, Ambion by Life Technologies, Carlsbad, CA, USA), quantified by a NANODROP spectrophotometer (NANODROP 2720, Thermal Cycler, Applied Biosystem, Waltham, MA, USA) and reverse transcribed using the Superscript Vilo cDNA synthesis kit (Life Technologies, Monza, Italy). Gene expression was evaluated by semiquantitative PCR analysis using the SYBR green PCR kit (QIAGEN GmbH, Hilden, Germany) in a Light Cycler 2.0 Instrument (Roche Diagnostics). Ten nanograms of cDNA were tested in duplicate for each sample. The protocol included a denaturation cycle at 95 °C for 15 min, 25 to 40 cycles of amplification (95 °C for 15″, the appropriate annealing temperature for each target, as detailed in Table 1, for 20″ and 72 °C for 20″) and a melting curve analysis to check for amplicon specificity. The mean threshold cycle was determined for each sample and used for the calculation of relative expression using the 2^−ΔΔCt^ method, with *GAPDH* as the reference gene and CTR as the calibrator [44].

### 2.6. Statistical Analysis

Data were analyzed using GraphPad Prism (GraphPad Software, version 9.0.0, San Diego, CA, USA). After having verified the homogeneity of variance with the Shapiro–Wilk test, a one-way ANOVA followed by Holm–Šídák’s multiple comparison post hoc and a two-way ANOVA followed by Holm–Šídák’s multiple comparison post hoc test were applied for SaOs-2 cytotoxicity and hBMSC bioactivity data, respectively. For all tests, the significance threshold was set at *p* < 0.05. AFM data were extracted by Gwyddyon software (version 2.66, Brno, Czech Republic). RMS and CA values were analyzed by Origin Software version 7.5 (OriginLab, Northampton, MA, USA).

## 3. Results

### 3.1. Surface Analyses

#### 3.1.1. Wettability

**Figure 2:**
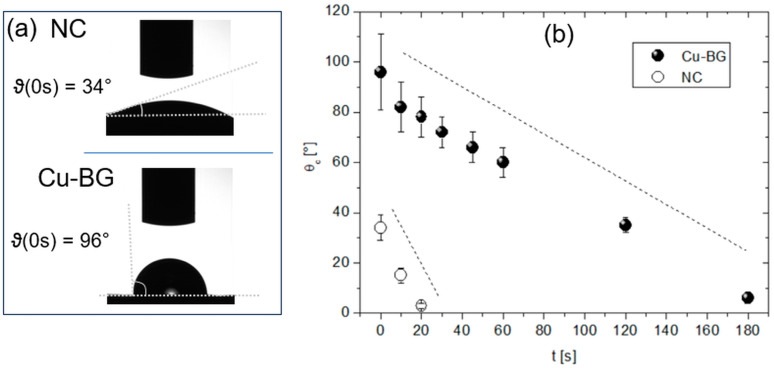
(**a**) Pictures of the water drops and indication of ϑc (0 s) for NC (**top**) and Cu-BG (**bottom**) samples. (**b**) Plot of ϑc vs. time for both samples. The dotted trend lines are provided as a visual reference.

Water CA (ϑc) was used to characterize the wettability of the NC and Cu-BG surfaces. From Figure 2a (top), initially, ϑc(0 s) ~ 34° (hydrophilicity) in the NC. However, due to its high porosity, the NC rapidly absorbs the drop, causing a decrease in ϑc to 0° after about 20 s (Figure 2b), while a longer time decay (~3 min) on the Cu-BG is required.

#### 3.1.2. Scanning Electron Microscopy

**Figure 3:**
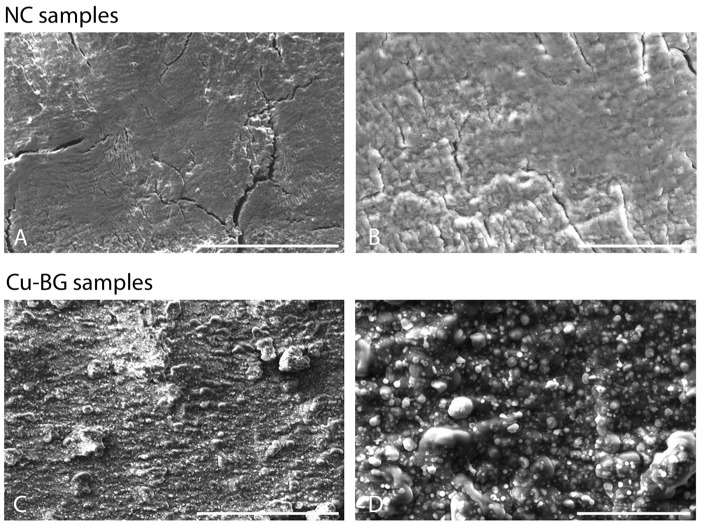
(**A**) 500× SEM image of the NC sample with visualization of crack lines and (**B**) 2000× detail on the same region. (**C**) The same as (**A**) taken on the Cu-BG sample. (**D**) The same as (**B**) for the Cu-BG sample. Scale bars: 250 µm for images (**A**,**B**) and 50 µm for (**C**,**D**).

In Figure 3, four SEM images recorded at different magnifications of both NC and Cu-BG samples are displayed. Long (100 s µm) cracks are visible across the NC surface (Figure 3A), together with smaller discontinuities, as in Figure 3B.

All these morphological features completely changed after the coating (Figure 3C,D), showing a grain-like topography and an apparently strong tendency to form aggregates.

#### 3.1.3. Atomic Force Microscopy

**Figure 4:**
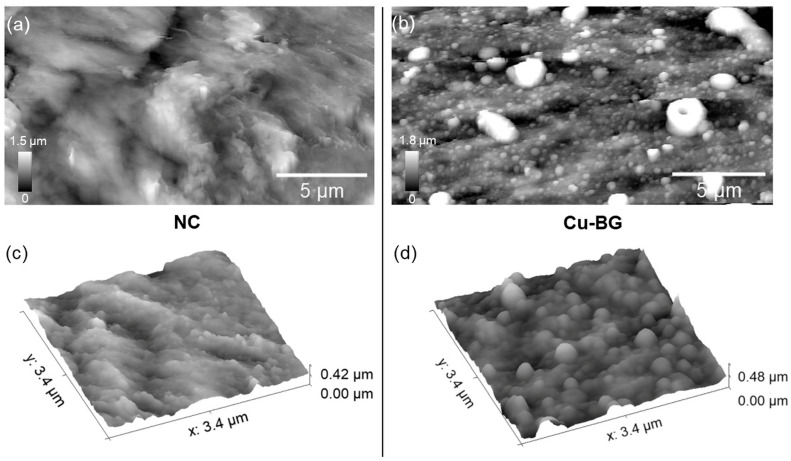
20 µm × 12.5 µm AFM micrographs of (**a**) NC and (**b**) Cu-BG samples; (**c**) 3.4 µm × 3.4 µm image of NC and (**d**) Cu-BG.

AFM was carried out to gain further insights into the modifications induced by the coating on the micro- and nanoscale bone structure. Representative AFM micrographs of the NC and Cu-BG samples are reported in Figure 4a and Figure 4b, respectively. Figure 4c,d display portions of the respective surfaces taken at higher magnification. In line with previous reports, two distinct contributions to bone architecture, namely the fibrous (collagenous) one and the particle-like (hydroxyapatite crystals) one, characterize the NC surface, as seen in Figure 4a,c. On the other hand, many different features characterize the Cu-BG surface, i.e., grain-like appearance, presence of micrometer-sized aggregates, and a wave-like surface that can be observed in Figure 4b,d.

**Figure 5:**
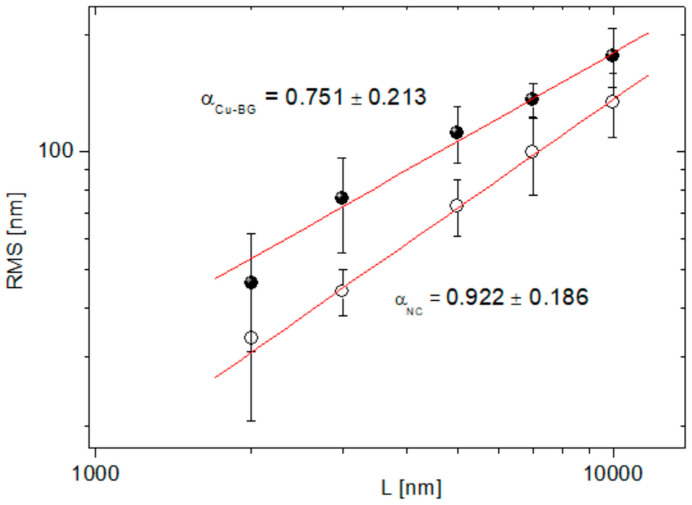
Log–Log plot of RMS vs. L for NC (circles) and Cu-BG (full dots), together with the corresponding linear regressions with slopes α_NC_ and α_Cu-BG,_ respectively (*p* < 0.0001).

As previously reported [45,46], films deposited by nanosecond PLD are usually characterized by micrometric droplets spread over a compact nano-granular structure. Likewise, under the experimental conditions described in the present manuscript, the micrometric droplets were embedded into the compact background. Consequently, an average film thickness of 500 nm was determined. This type of morphology is well-suited for biomedical applications since it facilitates osteoblast adhesion and proliferation [47]. Quantitative insights into surface modifications induced by the coating can be obtained by the analysis of the root-mean-square roughness (RMS) evolution within an opportune range of spatial scales. To this aim, in Figure 5, the Log–Log plot of RMS vs. L on both the NC and Cu-BG samples is reported. The relative amount of the uncertainties on the RMS values is determined by the high aspect ratio (up to 25%) of both the NC and Cu-BG surfaces. In addition to this, a different decay rate of the RMS vs. L was suggested by the different slopes α of the linear fittings (χ^2^ > 0.99) of the curves.

### 3.2. Cytotoxicity Evaluation

**Figure 6:**
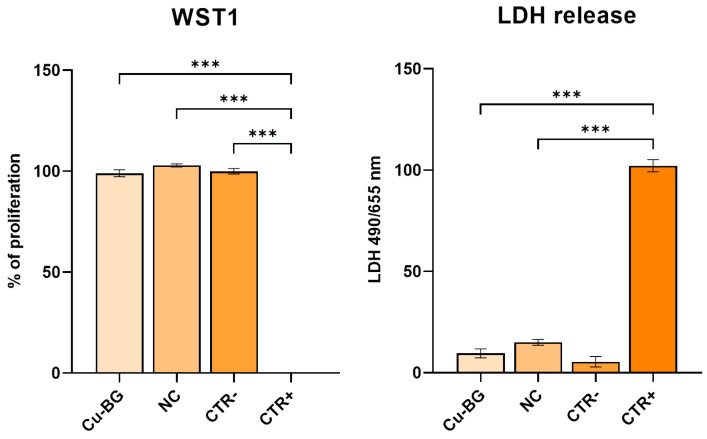
Barplots of WST1 test reported as viability percentage with respect to the negative control, considered equal to 100% (Mean ± SD, *n* = 3), and of LDH reported as the percentage of LDH release relative to CTR+ (Mean ± SD, *n* = 3). The mean value for CTR+ in the WST1 assay is 0.20 ± 0.09, indicating minimal viability. One-way ANOVA followed by Holm–Šídák’s multiple comparison test: ***, *p* < 0.001.

The quantitative evaluation of cytotoxicity was performed by measuring cell viability throughout the WST1 test, Neutral Red vital dye, and lactate dehydrogenase enzyme (LDH) release. The results are reported as a percentage of cell viability with respect to the negative control (CTR−; 100%) (Figure 6).

The graph shows that the cells in contact with the experimental samples had a viability rate of over 70%, which is the cut-off value for cytotoxicity when considering a CTR− of 100%, and no differences were detected among the experimental materials and the CTR−. Instead, significant differences were detected between the CTR+ and the Cu-BG, NC, and CTR− (*p* < 0.001). The results of the LDH measure were significantly different between the NC and CTR− (*p*= 0.006) and significantly lower than the positive control group (CTR+) (*p* < 0.001) (Figure 6).

**Figure 7:**
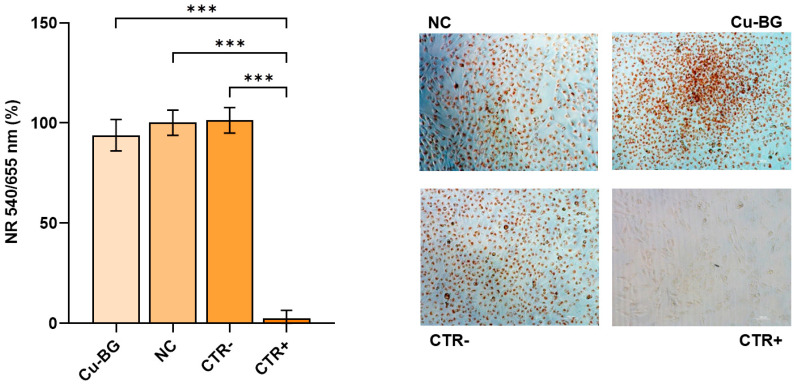
Results of neutral red quantification and microscopic images of stained cultures (Mean ± SD). One-way ANOVA followed by Holm–Šídák’s multiple comparison test: ***, Cu-BG, NC, and CTR− vs. CTR+ (*p* < 0.001). Magnification 10×; scale bar 100 μm.

Regarding the Neutral Red staining, live cells appear red-colored, while dead cells are transparent. Cellular distribution and density, as well as whether there are any areas of poor cell adhesion, are especially noticeable by means of the 4x magnification. The captured images reveal similar and regular colonization of the bottom well in the presence of the experimental samples and the negative CTR. Furthermore, there is no evidence of cell detachment. Conversely, the CTR+ presents no staining and a lower cell density due to detachment (Figure 7). No significant differences were observed between the samples and the CTR−, while a significantly lower percentage of cell staining was observed in the CTR+ compared to the NC, Cu-BG, and CTR− (*p* < 0.001) (Figure 7).

### 3.3. Bioactivity Evaluation

#### 3.3.1. hBMSC Viability

**Figure 8:**
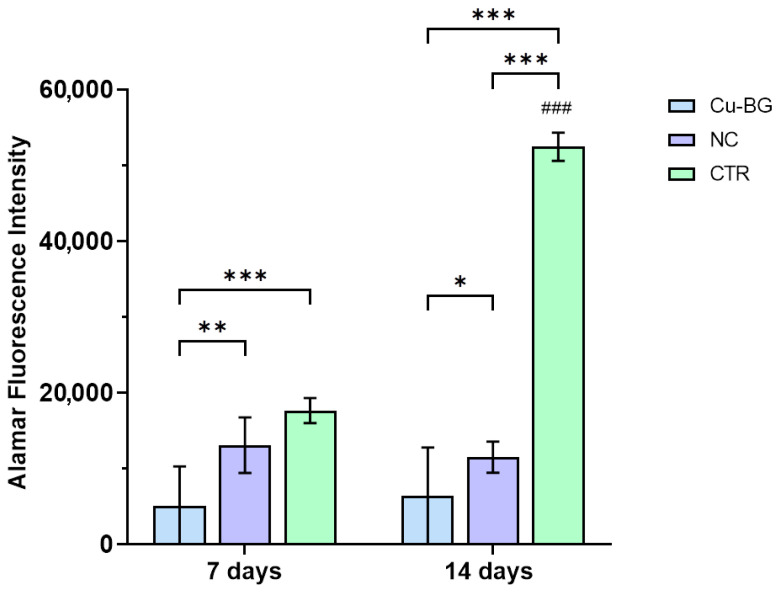
Barplots of hBMSC viability on Cu-BG, NC, and well plate (CTR) after 7 and 14 days of culture (Mean ± SD, *n* = 3). Two-way ANOVA test followed by Holm–Šídák’s multiple comparisons test: *, *p* < 0.05; **, *p* < 0.01; ***, *p* < 0.001; ###, CTR at 14 days vs. 7 days (*p* < 0.001).

The results for the cell viability of the hBMSCs seeded on the NC and Cu-BG samples or the well plate (CTR) are presented in Figure 8. The CTR group exhibited significantly higher values than the cells seeded on the Cu-BG and NC samples at 14 days (*p* < 0.001) and between 7 and 14 days (*p* < 0.001). Additionally, CTR viability was significantly higher at 7 days compared to the Cu-BG (*p* < 0.001) samples. At each time point, the dye reduction values are significantly higher in the NC samples compared to the Cu-BG samples (7 days: *p* = 0.003; 14 days: = 0.03).

#### 3.3.2. hBMSC Osteogenic Differentiation

**Figure 9:**
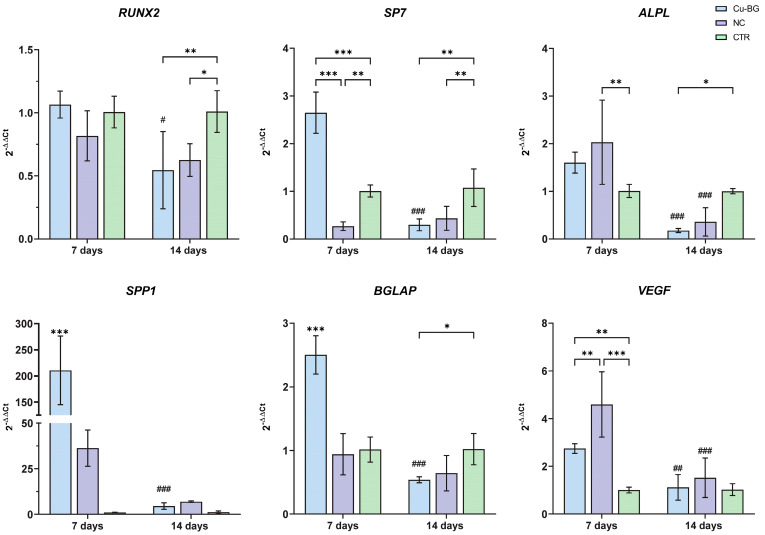
Gene expression analysis. Relative gene expressions of *RUNX2*, *SP7*, *ALPL*, *SPP1*, *BGLAP*, and *VEGF* after 7 and 14 days of culture on Cu-BG and NC reported as fold changes with respect to cells grown on well plate (CTR), considered equal to 1 (Mean ± SD, *n* = 4). Two-way ANOVA test and adjusted Holm–Šídák’s multiple comparisons test: *, *p* < 0.05; **, *p* < 0.01; ***, *p* < 0.001; 14 days vs. 7 days for the same material: #, *p* < 0.05; ##, *p* < 0.01; ###, *p* < 0.001.

Figure 9 reported the relative gene expressions of *RUNX2*, *SP7*, *ALPL*, *SPP1*, *BGLAP*, and *VEGF*. The expressions of the *RUNX2*, *SP7,* and *ALPL* genes decrease over time (from 7 to 14 days) in cells grown on the Cu-BG coated materials (*RUNX2*, *p* = 0.01; *SP7* and *ALPL*, *p* < 0.001). *RUNX2* expression is higher at 14 days in the control samples compared to the Cu-BG samples (*p* = 0.007) and NC samples (*p* = 0.02). The *ALPL* gene shows significant decreases in the NC and Cu-BG samples between 7 and 14 days, and higher expression in the NC samples at 7 days compared to the control (*p* = 0.005). However, its expression decreases significantly over time (*p* < 0.001). At 7 days, the SP7 gene expression is significantly higher in the Cu-BG samples compared to the NC (*p* < 0.001) and CTR (*p* < 0.001). Furthermore, expression levels are higher in the CTR compared to NC samples (*p* = 0.01) at 7 days, while at 14 days, the control shows higher levels than the Cu-BG (*p* = 0.002) and NC (*p* = 0.007) samples. At 7 days, *SPP1* and *BGLAP* expression levels are significantly higher in the Cu-BG samples compared to both the NC and CTR groups (*p* < 0.001). However, their expression decreases significantly over time (*p* < 0.001). Additionally, at 14 days, *BGLAP* expression is significantly lower in the Cu-BG samples compared to the CTR group (*p* = 0.04). Finally, VEGF expression was significantly higher in the NC samples compared to the CTR control (*p* < 0.001) and Cu-BG (*p* = 0.03) at 7 days and in the Cu-BG compared to CTR (*p* = 0.03). Furthermore, its values decrease significantly between 7 days and 14 days (*p* < 0.001) in the NC samples. Finally, Cu-BG expressed significantly higher values at 7 days compared to 14 days (*p* = 0.08).

## 4. Discussion

In the bone regeneration field, new bioactive materials are constantly being developed. However, to our knowledge, this study is the first one in which a bioactive material has been deposited on the surface of allogeneic bone grafts to enhance their biological properties. Specifically, our in vitro model is based on human freeze-dried cortical bone tissue shaped into cylinders with reproducible dimensions using CNC machining and then coated with a Cu-doped BG coating deposited by the PLD technique. Cylinder-shaped bone samples were chosen for this model due to systematic reproduction of sample parameters and the possibility of being placed vertically on the smooth horizontal surface of polystyrene culture plates, similar to how common 3D-printed scaffolds are used in tissue-engineering protocols [48,49], but with the advantage of meeting the structural, mechanical, and osteoconductive requirements of native bone. Furthermore, a slight modification of a PLD deposition protocol developed in a previous work [26] has been adopted to coat the surface of allogeneic freeze-dried bone—a common tissue bank product primarily used as a scaffolding matrix for various clinical applications—with the same target material [50].

The CA analysis of the NC reflects its well-known tendency towards hydrophilicity, which is essential for bone metabolism and its reparative mechanisms. Remarkably, although the presence of the coating significantly altered the initial value of ϑc and its time evolution (absorption rate), the hydrophilic character of the surface was still preserved.

It is well established that the sensitivity of bone cells is not solely dependent upon chemical surface properties, but also on surface geometry and topography, which exert a profound influence on cell adhesion, proliferation, and differentiation [51]. As highlighted by the SEM results, the deposition resulted in a densely and uniformly coated surface, which altered the pristine bone surface morphology across different length scales (filling of crack lines and pits, substitution of asperities), indicating a non-conformal coating deposition [52]. Such substitution is confirmed at the AFM scale, where an overall increase in the RMS can be observed for the Cu-BG, at least at scales smaller than 10 µm (Figure 5), as a result of the increase of the topographic fluctuations along the z-axis. The continuity of the surface, compared to the porosity of pristine bone, together with the metal-like nature of the coating, is more likely the microscopic origin of the different CA behaviors observed in the two batches of samples.

The in vitro results offer valuable insights into the cytocompatibility and bioactive properties of this material designed for bone tissue contact. The use of Saos-2 cell line and primary hBMSCs in this study was carefully designed to align with the specific objectives of each biological evaluation. The Saos-2 osteoblast-like cell line was chosen for cytotoxicity testing in accordance with UNI EN ISO 10993-5:2009 [43], which recommends the use of well-characterized, stabilized cell lines to ensure reproducibility and robustness in cytotoxicity assays. Saos-2 cells, being widely used in biocompatibility studies, provide a standardized model to assess toxicity to biomaterials in contact with bone tissue. Furthermore, differently from other cell lines usually utilized, they are also prone to mineralization [53,54,55]. On the other hand, hBMSCs were employed to evaluate the osteogenic potential of the bioactive glass coating, as they better mimic the in vivo behavior of osteoprogenitor cells. hBMSCs retain the ability to differentiate into osteoblasts, making them a more physiologically relevant model for assessing the material’s osteoinductive properties. Several studies have confirmed that hBMSCs represent a suitable model for bone regeneration studies due to their ability to respond to bioactive stimuli and differentiate into bone-forming cells [56,57].

Evaluating cytotoxicity through various parameters, such as cell viability and LDH release, revealed that Cu-BG did not exhibit significant cytotoxic effects and did not compromise cell viability compared to the NC samples. Moreover, the analysis of cell morphology using the Neutral Red dye demonstrated comparable cell distribution and reduced cell detachment in both sample groups when compared to the positive control (cells treated with 1% phenol solution in DMEM), thus confirming their biocompatibility. The alamarBlue™ test highlighted different behaviors among the groups. The hBMSCs seeded on plastic wells (CTR) showed a significant increase in viability over time in comparison to the Cu-BG samples. Significant differences were observed between the Cu-BG and NC samples at both experimental time points. Specifically, the Cu-BG exhibited decreased viability in comparison to the NC samples, which consistently demonstrated the highest viability levels.

This difference may be attributed to the combined action of two phenomena: an increase in cell commitment towards the osteoblastic phenotype enhanced by the coating, and the effect of the release of Cu^2+^ ions, resulting in a slight cell cytotoxicity. However, this is unlikely because our analysis of cell viability in the cytotoxic assays over a 2-day culture period showed that it remains well above 90%. Another possible explanation could be the one reported by Rodriguez et al. [58]. They assessed a concentration-dependent decrease in the proliferation rate of mesenchymal stem cells (MSCs) cultured in media containing an increased concentration of copper and explained this finding by noting that copper leads to an increase in the size of the cells. Consequently, the cells would reach confluency with a lesser number of cells, and cell proliferation would be inhibited by cell-to-cell contact. However, it is probable that copper affects cell proliferation by influencing one or more specific stages of the cell cycle. The process of bone formation involves several distinct phases, including proliferation, maturation of the extracellular matrix, and mineralization. These phases are coordinated by a multitude of molecular pathways. During osteogenesis, the initial downregulation of the proliferation phase triggers the synthesis of bone matrix proteins. In this context, the transcription factor *RUNX2*, in conjunction with its downstream effector *SP7*, plays a pivotal role in directing the expression of essential genes for osteoblast differentiation and bone matrix production [59,60]. The observed increase in gene expression of the *RUNX2* and *SP7* genes, which is significantly higher at 7 days than at 14 days, particularly in the Cu-BG samples, corroborates this commitment trend. Previous studies have suggested that Cu^2+^ ions could induce the differentiation of mesenchymal stem cells and osteoblastic cells [58,61]. The analysis of gene expression related to the osteogenic differentiation of hBMSCs revealed significant differences between the experimental groups. Specifically, the expressions of the *ALPL*, *BGLAP*, and *SPP1* genes were higher in the experimental samples coated with doped bioactive glass compared to those of the cells grown on plastic dishes. These results are consistent with those of Wu et al. [62], who observed an increase in the expression of these bone-related gene expressions in hBMSCs cultured on (Cu)-containing mesoporous bioactive glass (Cu-MBG) scaffolds at 7 days. This suggests a potential positive effect on early osteogenic stimulation, which is important for promoting new bone formation.

To extend the investigations to other functions of Cu-doped bioactive glass, the expression of vascular endothelial growth factor (*VEGF*) was observed. Copper ions have been recognized for decades for their ability to stimulate angiogenesis and enhance the development of blood vessels, promoting the formation of a vascularized structure, which is strictly a prerequisite for new bone ingrowth, particularly in compact cortical bone [62,63,64]. The expression of *VEGF* was significantly higher in the experimental samples compared to the control samples, particularly at 7 days compared to 14 days, suggesting a stimulation of vasculogenesis, which is highly desirable for bone tissue repair. Indeed, in the absence of adequate nourishment through vascular pathways, cells undergo necrosis, leading to the failure of any bone substitute to adequately replace the damaged tissue. Vascularized bone plays a critical role in supporting weight, addressing significant bone depletion, and facilitating accurate healing at the site of injury. Nevertheless, it is essential to recognize the need for further investigation in this area. In particular, conducting additional assays, such as the tube formation assay, is crucial for a more detailed elucidation of the angiogenic potential. The comprehensive molecular biology data suggest that the Cu-BG coating provides an initial osteogenic and angiogenic stimulus to BMSCs that should be further explored with longer experimental times. BGs stand out as vital biomaterials in scaffold-centered approaches for bone tissue engineering. Their remarkable osteoinductive and angioinductive characteristics surpass those of alternative biomaterials, making them a pivotal choice in the field. Typically, bone replacement materials are assessed with additional elements such as copper (Cu^2+^) [65], zinc (Zn^2+^) [66], magnesium (Mg^2+^) [67], and strontium (Sr^2+^) [68]. Among these, Cu^2+^ is particularly recognized for its significant role in fostering both osteogenesis and angiogenesis [69] and antibacterial properties [70,71].

The study is constrained by several limitations. First, in this work, it was not possible to evaluate the adhesion strength of the prepared coatings due to the specificity of our substrates—i.e., human cortical bone cylinders—which are not metallic or non-metallic surgical implants. However, usually, PLD films are characterized by a high adherence to the substrate. For example, it has been reported that adhesion strength over 15 MPa has been achieved for hydroxyapatite coatings deposited on TiN substrates [72]. Nevertheless, further studies, also related to the choice of the best adhesion strength test for a bone tissue surface (e.g., scratch, shear, or pull-out bonding tests), are needed to overcome drawbacks related to degradation or separation of the Cu-BG from the bone surface over time, which could alter the construct’s functionality. Second, the lack of an analysis of copper release in the culture medium would have enabled a more accurate assessment of the observed trend in hBMSC vitality. However, based on the results of our numerous previous studies on similar coatings for titanium implants and various copper-doped bone substitute materials, it is well established that the amount of copper used in this work does not produce any significant cytotoxic effect [26,65,70,71]. Furthermore, a direct evaluation of mineral deposition and matrix formation, for example, through Alizarin Red staining, was not conducted. Similarly, the assessment of protein secretion, crucial for bone matrix formation, was limited. Further studies are necessary to fully elucidate the mechanism of action of these materials and to confirm their effectiveness in preclinical and clinical applications for bone regeneration. Nevertheless, to the best of our knowledge, this is the first study investigating the biological cues of allogeneic cortical bone tissue coated with a bioactive material. Our aim was to develop a composite biomaterial that combines the osteoconductive properties and biomechanical competence of cortical bone with the osteogenic and angiogenic capacities of Cu-doped bioactive glass. In this regard, these preliminary results may pave the way for developing an innovative class of bone-based products distributed by tissue banks.

## 5. Conclusions

In the present study, the deposition of Cu-BG using the PLD technique on human cortical bone results in a densely and uniformly coated surface with enhanced microscale roughness compared to uncoated bone, while water CA measurements show a total absorption of the drop on both uncoated and coated surfaces. Moreover, the synergistic effect of a Cu-containing BG and an allogeneic bone graft on cellular response in terms of growth and osteo-angiogenic differentiation at an early stage was studied. Specifically, Cu-BG deposition demonstrated no cytotoxic effects and adequate biocompatibility, as shown by WST1, LDH, and Neutral Red analyses, in which no significant differences were detected on the NC and Cu-BG samples. Moreover, Cu-BG deposition on allogeneic bone grafts positively influences the hBMSCs seeded on board, as shown by the up-regulation of *RUNX2*, *SP7*, *ALPL*, *BGLAP*, and *SPP1*, which are involved in the osteogenic commitment, and *VEGF*, which is involved in pro-angiogenic differentiation. Therefore, the bioactive, osteogenic, and angiogenic properties shown by the composite bone-based material developed with the protocol presented here make it a promising candidate for a potential translation into future clinical applications related to bone defect repair and reconstruction, spinal fusion, or non-union fracture healing. Nevertheless, further studies are required to confirm its efficacy for bone repair and regeneration procedures.

## Figures and Tables

**Table 1:** Specifications of primer used for the gene expression analysis.

Gene	Primer	Amplicon Length	Annealing Temperature
*GAPDH*	QuantiTect Primer Assay (Qiagen) Hs_GAPDH_1_SG	95 bp	55 °C
*RUNX2*	Forward: CTTCACAAATCCTCCCCAAGT Reverse: AGGCGGTCAGAGAACAAAC	212 bp	60 °C
*ALPL*	QuantiTect Primer Assay (Qiagen) Hs_ALPL_1_SG	110 bp	55 °C
*SP7*	QuantiTect Primer Assay (Qiagen) Hs_SP7_1_SG	120 bp	55 °C
*BGLAP*	QuantiTect Primer Assay (Qiagen) Hs_BGLAP_1_SG	90 bp	55 °C
*VEGF*	QuantiTect Primer Assay (Qiagen) Hs_VEGFA_6_SG	99 bp	55 °C
*SPP1*	QuantiTect Primer Assay (Qiagen) Hs_SPP1_1_SG	115 bp	55 °C

## Data Availability

Data pertaining to cell viability and gene expression are available upon request from the corresponding author.
